# Strategies for biofilm optimization of plastic-degrading microorganisms and isolating biofilm formers from plastic-contaminated environments

**DOI:** 10.1093/sumbio/qvae012

**Published:** 2024-05-17

**Authors:** Adam McFall, Scott A Coughlin, Gary Hardiman, Julianne Megaw

**Affiliations:** School of Biological Sciences, Queen's University Belfast, 19 Chlorine Gardens, Belfast, BT9 5DL Northern Ireland, United Kingdom; School of Biological Sciences, Queen's University Belfast, 19 Chlorine Gardens, Belfast, BT9 5DL Northern Ireland, United Kingdom; School of Biological Sciences, Queen's University Belfast, 19 Chlorine Gardens, Belfast, BT9 5DL Northern Ireland, United Kingdom; School of Biological Sciences, Queen's University Belfast, 19 Chlorine Gardens, Belfast, BT9 5DL Northern Ireland, United Kingdom

**Keywords:** biofilms, environmental microbiology, isolation, fungi, biodegradation of plastics, high density polyethylene, low density polyethylene, polyethylene terephthalate

## Abstract

The perpetual disposal of plastic waste, combined with ineffective waste management strategies, has resulted in widespread environmental plastic pollution. Microbial plastic biodegradation represents an emerging solution to this problem. However, biodegradation studies tend to overlook the fundamental prerequisite of initial surface colonization via biofilm formation. This study had two independent but connected aims relating to plastic surface colonization by microorganisms: to enhance biofilm formation by known plastic degraders, with translational potential for improved plastic degradation, and to isolate microorganisms from microplastic contaminated environments with the ability to colonize plastic surfaces. Planktonic and biofilm responses to diverse carbon and energy sources were investigated over 7 days, using *Bacillus subtilis* 168, *Fusarium solani* (Martius) Saccardo, *Ideonella sakaiensis* 201-F6, *Pseudomonas putida* KT2440, and *Rhodococcus ruber* C208. This enabled optimal conditions for biofilm formation by each strain to be determined. In parallel, environmental samples containing synthetic or natural polymeric substances (anaerobic digestate, landfill leachate, and microplastic contaminated compost) were incubated with polyethylene and polyethylene terephthalate films, to isolate microorganisms capable of colonizing their surfaces. This yielded eight bacterial isolates from three genera: *Bacillus, Lysinibacillus*, and *Proteus*. These genera contain species that have been shown to degrade plastics and other recalcitrant synthetic polymers, demonstrating the success of our approach. This study also suggests that discrete plastic types may create different ecological niches which can be exploited by unique bacterial colonizers. Our findings underscore the importance of considering plastic colonization by microbial biofilms in the context of their biodegradation.

**Sustainability Statement**

This study provides valuable insights into plastic surface colonization by microorganisms, which is a fundamental prerequisite for their biodegradation. We have identified improved culture conditions to enhance biofilm formation by known plastic degraders, in addition to outlining an approach for the isolation of biofilm formers from plastic-contaminated environments. This work specifically addresses the United Nations Sustainable Development Goals 11 (Sustainable Cities and Communities) and 12 (Responsible Consumption and Production).

## Introduction

Single-use plastics have proliferated throughout modern civilization and represent a key marker of the Anthropocene era. The term “plastic” describes many high molecular weight polymers derived from petroleum hydrocarbons (Bahl et al. 2021, Verschoor et al. 2022). Their versatility, durability, and sterility has enabled limitless applications throughout agriculture, commerce, and industry (Gilan et al. 2004, Maurya et al. 2020, Zeenat et al. 2021). However, desirable characteristics of plastics such as impermeability, hydrophobicity, and stability have created a significant barrier to their natural degradation (Maurya et al. 2020, Amobonye et al. 2021). Furthermore, indiscriminate disposal of plastics coupled with their environmental persistence has resulted in widespread pollution, from terrestrial soil to freshwater and marine ecosystems (Gilan et al. 2004, Danso et al. 2019, Kotova et al. 2021, Usman et al. 2022). Globally, 7 billion tonnes of plastic waste were produced between 1950 and 2017, with the vast majority (76%) being abandoned in landfills or infiltrating natural environments (Geyer 2020). Specifically, polyethylene (PE) and polyethylene terephthalate (PET) represent significant contributors to worldwide plastic production (PE = 36% and PET = <10%) and pollution, with their extensive lifespans of 600 and 450 years, respectively (Geyer et al. 2017, Mohanan et al. 2020).

While they may not appear inherently harmful, the environmental accumulation of plastics, microplastics (MPs = <5 mm), and nanoplastics (NPs =<0.1 µm) has resulted in a myriad of environmental consequences (Danso et al. 2019, Iroegbu et al. 2021). Plastics have been described as multifaceted stressors, acting as vectors of persistent organic pollutants, heavy metals, reproductive toxins, endocrine disruptors, and mutagenic agents (Schmaltz et al. 2020, Zeenat et al. 2021, Verschoor et al. 2022). Furthermore, plastics are known to entangle marine life and block the digestive systems of various animal species, increasing mortality (Maurya et al. 2020, Yang et al. 2021).

Significantly, current waste management strategies that rely on landfill storage, incineration, or mechanical recycling are either ineffective or unsustainable (Verschoor et al. 2022). Therefore, exploring alternatives such as microbial biodegradation is imperative to addressing the exponential threat of plastic pollution (Ru et al. 2020). Biodegradation refers to the decomposition of synthetic polymers via microbial action, and occurs in four stages: biodeterioration, biofragmentation, assimilation, and mineralization (Jacquin et al. 2019, Amobonye et al. 2021, Zeenat et al. 2021). Biodegradation of plastics is possible due to the extraordinary metabolic plasticity of microorganisms, which have evolved to exploit unique ecological niches, including a variety of anthropogenic compounds (Kotova et al. 2021). Diverse microbial communities colonizing environmental plastic debris have been specifically referred to as the “plastisphere” (Zettler et al. 2013). Theoretically, plastics represent untapped reservoirs of carbon, hydrogen, and oxygen to support microbial metabolism (Ekanayaka et al. 2022). Moreover, microbial populations already possess enzymatic and metabolic pathways to degrade existing natural polymers such as cellulose and lignin, potentially overlapping with unexplored plastic degradation pathways (Maurya et al. 2020, Ekanayaka et al. 2022). Enzymatic and metabolic pathways associated with PE and PET biodegradation have been identified in several microbial species (Jacquin et al. 2019, Maurya et al. 2020) including *Arthrobacter, Bacillus, Pseudomonas, Rhodococcus*, and *Streptomyces* species, which represent prominent bacterial taxa in the plastics biodegradation literature (Sivan et al. 2006, Vimala and Mathew 2016, Espinosa et al. 2020, Han et al. 2020), in addition to fungal species including *Fusarium solani pisi* and *Humicola insolens* (Alisch-Mark et al. 2006, Danso et al. 2019, Tournier et al. 2020, Ekanayaka et al. 2022).

Whilst a variety of microbial species display an intrinsic ability to degrade plastics, a major bottleneck results from specific properties of plastics, including surface hydrophobicity, topography, and crystallinity, which significantly prevent microbial interactions (Danso et al. 2019, Zeenat et al. 2021). Fundamentally, plastic biodegradation is defined as a “surface erosion” process; therefore, direct microbial attachment is necessary for effective biodegradation (Sivan 2011, Wilkes and Aristilde 2017, Amobonye et al. 2021, Lens-Pechakova 2021). Specifically, PE and PET are densely cross-linked polymers with limited accessibility for enzymatic attack, solely confined to the external surface (Jacquin et al. 2019). Therefore, overcoming the initial barrier of surface colonization through biofilm enhancement is a viable strategy for improved microbial plastic biodegradation.

Biofilms are mixed polymicrobial communities, consisting of cells encapsulated within a self-produced extracellular polymeric substances (EPS) matrix, where they adhere to each other and usually also to a surface (Donlan 2002, Flemming et al. 2016). Biofilm development occurs in sequential stages including initial attachment, microcolony formation, biofilm maturation, and detachment (Crouzet et al. 2014). Therefore, initial surface attachment by early microbial colonizers is fundamental to successive biofilm developmental stages (Grinberg et al. 2019). It is widely acknowledged that plastic biodegradation is preceded by biofilm formation, which enhances microbial and enzymatic interactions through direct surface contact with the polymer (Plakunov et al. 2020, Kotova et al. 2021, Chattopadhyay 2022). Importantly, biofilms create an external “digestive system” through the entrapment of extracellular enzymes within the EPS. Enhancing biofilm formation of plastic-degrading bacteria has been shown to increase both the proximity of plastic-degrading enzymes at the plastic surface, and their local concentration, thereby improving the overall rate of biodegradation (Costa et al. 2018, Howard and McCarthy 2023). Beneficially, entrapment of enzymes in the EPS can also increase their resistance to dehydration, thermal denaturation, and proteolysis (Flemming et al. 2016).

In the present study, we investigated the impact of supplemental carbon and energy sources on both planktonic and biofilm growth of microbial strains known to be capable of plastic degradation (specifically of PE and PET). In parallel, microplastic polluted environments were investigated to isolate microbial strains capable of plastic surface colonization, in order to identify candidate strains with biodegradation potential. We hypothesized that (1) there would be a significant difference in the planktonic growth of each strain when cultured using various carbon and energy sources over 7 days; (2) there would be a significant difference in the biofilm development of each strain when cultured using various carbon and energy sources over seven days; and (3) biofilm-forming microbial strains within microplastic polluted environments would colonize discrete plastic polymers. Subsequently, endpoint (7 day) biofilm results were used to identify “optimal” nutrient conditions which may be applied to enhance biofilm formation of the selected strains in biodegradation studies, and we validated an isolation strategy to selectively obtain microorganisms from environmental samples with an ability to colonize plastic surfaces.

## Materials and methods

### Test strains and culture conditions

The known plastic degraders *Bacillus subtilis* 168, *F. solani* (Martius) Saccardo, *Pseudomonas putida* KT2440, and *Rhodococcus ruber* C208 were purchased from the DSMZ culture collection. *Ideonella sakaiensis* 201-F6 was obtained from the NITE Biological Resource Centre (NBRC). Table S1 outlines the routine culture conditions for each strain.

### Materials and Reagents

High Density Polyethylene (HDPE) (Thickness: 0.01 mm), Low Density Polyethylene (LDPE) (Thickness: 0.025 mm) and PET (Thickness: 0.013 mm) films were purchased from Goodfellow Cambridge Ltd. All equipment, chemicals, and reagents used in this study, and their sources, are outlined in Table S2. All reagents and culture media were prepared using reverse osmosis (RO) water.

### Microtiter assays for planktonic growth and biofilm development

All assays were conducted in M9 minimal salts medium supplemented with various carbon and energy sources, which represented either common microbial growth medium components or plastic monomers (Table 1). Autoclaved stock solutions of each nutrient were adjusted with sterile RO water to 2 × the required initial concentrations (1.0 and 0.5% w/v). Each nutrient solution was then added to eight replicate wells in a 96-well microtiter plate (100 µL/well; Fig. S1). All test strains were prepared by diluting 24–48 h liquid cultures 1:50 in modified 2 × concentrated M9 medium. Test wells were inoculated with 100 µL/well, to generate the final concentrations of 0.50% or 0.25% w/v of the carbon and energy sources, and 1:100 culture dilution. Uninoculated control wells for each test condition were also included in all assay plates (eight replicates). Triplicate plates were prepared for each set of test conditions, which were subsequently incubated for 1, 3, or 7 days (30˚C and 100 rpm). Post incubation, planktonic growth was quantified via absorbance (OD_600_) measurements using a CLARIOstar plate reader. Biofilm development of each strain was then evaluated for the same plates, following a similar procedure as outlined by O'Toole (2011), with minor modifications. Culture media were discarded from the wells and plates were rinsed by submerging in water three times, to eliminate loosely adhered cells. Crystal violet (CV) solution (0.1% w/v) was then added (200 µL/well) to stain any adherent biomass. The solution was discarded after 15 min, and plates were submerged in water to remove excess CV. Plates were then left for 24 h to allow any remaining water to evaporate. Acetic acid (30% v/v) was then added (200 µl/well) to solubilize the CV within the wells. Finally, biofilm development was quantified via absorbance (550 nm) using a CLARIOstar plate reader. Biofilm development and planktonic growth were calculated by subtracting the control well values from test well results.

**Table 1.** Summary of carbon and energy sources, concentrations, and incubation periods applied to evaluate biofilm formation of known plastic-degrading microorganisms.

Modified M9 medium (×2 concentration) (g L^−1^)	Carbon/energy source	Concentrations (% w/v)	Incubation period (days)
KH_2_PO_4_ (6.0) NaCl (1.0) Na_2_HPO_4_ (13.2) NH_4_Cl (2.0) MgSO_4_.7H_2_O (4.0)	Peptone (PPT) Yeast extract (YST) Meat extract (MEAT) Malt extract (MALT) Trypticase Soy Broth (TRP) Casamino acids (CAA) Sodium acetate (SA) Ethylene glycol (EG)*	0.50 and 0.25	1, 3, and 7

* Ethylene glycol solution was used as a PET monomer.

### Isolation of biofilm formers from environmental samples

Three environmental samples were collected with the aim of isolating microbial colonizers of HDPE, LDPE, and PET films. Sample selection was based on the presence of synthetic or natural polymeric substances within the ecosystem, and included agricultural anaerobic digestate, landfill leachate, and microplastic contaminated compost (Table 2).

**Table 2.** Overview of environmental sampling locations and rationale.

Sample	Source	Rationale
Anaerobic digestate	Agri-Food and Biosciences Institute, (Hillsborough, Northern Ireland)	Anaerobic digestate contains a diverse consortium of bacterial, archaeal, and fungal species metabolising mixed feedstocks, ranging from animal manure to agricultural mulch films and crop residues. This provides a variety of natural polymers and lignocellulosic material, which could promote microorganisms with unique hydrolase, esterase, or cutinase enzymes, applicable to plastic biodegradation.
Landfill leachate	Mullaghglass Landfill (non-hazardous) Regeneration Project (Lisburn, Northern Ireland)	Landfill leachate contains an abundance of mixed synthetic polymers (mixed municipal waste), variety of microplastics and unique microbial populations (Meyer-Dombard et al. 2020). The rationale for LL sampling is supported by the similar study by Yoshida et al. (2016) in which the PET-degrading *I. sakaiensis* 201-F6 was isolated along the periphery of a plastic bottle recycling facility in Osaka, Japan.
Microplastic contaminated compost	Commercial compost (provided by Dr Paul Williams, School of Biological Sciences, Queen's University Belfast)	Commercial compost samples were characterized internally at QUB as having mixed microplastic contamination. In theory, existing exposure to synthetic polymers within the terrestrial soil microbiome has the potential to shape microbial population demographics by enriching for microorganisms possessing enzymatic and metabolic pathways associated with plastic biodegradation.

A screening procedure was developed to selectively culture microbial strains with the ability to colonize plastic films (HDPE, LDPE, and PET). The selected environmental samples (anaerobic digestate/landfill leachate, 10 mL; contaminated compost, 10 g) and M9 minimal medium (10 mL, 2 × concentration) were added at a 1:1 ratio into sterile 50 mL centrifuge tubes (total volume 20 mL). Plastic films were cut into 20 × 20 mm (10.00 ± 1.00 mg) pieces, sterilized using 70% ethanol, and aseptically transferred into each tube. Contaminated compost was cultured under aerobic conditions, and anaerobic digestate and landfill leachate were cultured under anaerobic conditions in serum bottles. For anaerobic cultures, 1 mL of resazurin (0.1% w/v) was added to 10 mL of M9 minimal medium, brought to the boil and sparged with CO_2_ gas with cysteine-HCl (0.8% w/v). The reduced M9 solution was transferred into serum bottles (UV sterilized) containing the plastic films and sealed. The environmental samples were then aseptically transferred into the corresponding serum bottles via syringe. All environmental samples were incubated at 28˚C, 100 rpm, for 42 days. Following incubation, films were aseptically removed and repeatedly vortexed (30 seconds) in sterile M9 medium (10 mL) to remove any loosely adhered cells. Plastic films were then streaked onto Luria-Bertani agar plates and cultured at 28˚C until visible colonies developed. Colonies were re-streaked to ensure purity.

Isolates were identified using an Autobio Autof MS1600 matrix-assisted laser desorption ionization time-of-flight (MALDI-TOF) mass spectrometry (MS) microbial identification system (Chirus Ltd.). This method generates a unique proteomic fingerprint directly from microbial biomass, enabling rapid, comprehensive, and inexpensive microbial identification (Alizadeh et al. 2021). The direct coating method was used, whereby fresh colonies were transferred onto a target plate using a sterile toothpick, and each sample was then overlaid with 1 µL of matrix solution. The target plate was dried at 40˚C for 1 min, and the plate was inserted into the instrument. Spectral data were collected by Autof Acquirer version 2.0.196 and analyzed using Autof Analyzer version 2.0.14, with each isolate being identified using the included spectral database, version 1.1.18. The manufacturer's interpretation criteria were applied, with scores >9 indicating reliable species-level identification, scores 6–9 indicating reliable genus-level identification, and scores <6 defined as “unreliable.”

### Statistical analysis

Statistical analysis and data visualization were completed using R Studio version 4.1.1 (2021–08-10). Data generated during microtiter assays was evaluated using summary statistics, including the Shapiro-Wilk test and Bartlett's test to identify distribution patterns and homogeneity of variance. Non-parametric tests including the Kruskal-Wallis and post-hoc Dunn's test were applied for bacterial and fungal biofilm assays due to non-normal distribution and lack of homogeneity of variance. Prior to downstream data analysis, peripheral wells (rows A and H) of microtiter assays were excluded due to a heterogenous pattern of cell growth caused by the “edge effect” phenomenon (Mansoury et al. 2021). Day 7 biofilm data were evaluated to inform “optimal” nutrient selection based on the greatest biofilm formation (measured by absorbance at 550 nm) and statistical significance (*P*-value = <0.05).

## Results

### Planktonic and biofilm growth of known plastic degraders

**Figure 1.**
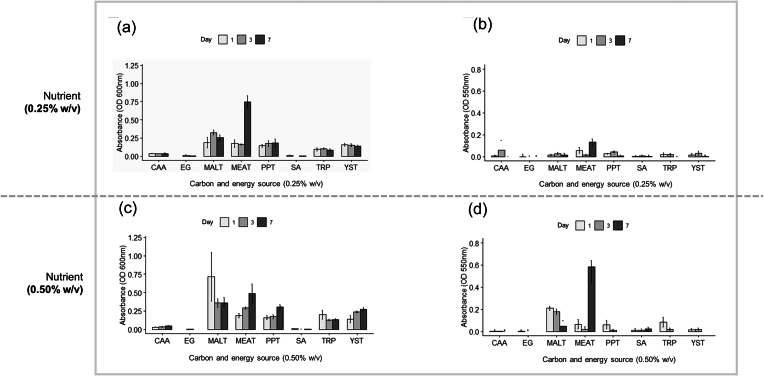
*Bacillus subtilis* 168 planktonic growth and biofilm formation measured by absorbance (600 and 550 nm, respectively) at various incubation periods (day 1, 3, and 7) to compare different carbon/energy sources and concentrations. Day 7 biofilm formation results were then examined to identify the “optimal” nutrient which displayed significant and long-term biofilm formation. (a) *B. subtilis* 168 planktonic growth using 0.25% w/v nutrient concentrations. (B) *B. subtilis* 168 biofilm response using 0.25% w/v nutrient concentrations. (c) *B. subtilis* 168 planktonic growth using 0.50% w/v nutrient concentrations. (d) *B. subtilis* 168 biofilm response using 0.50% w/v nutrient concentrations. Abbreviations: casamino acids (CAA), ethylene glycol (EG), malt extract (MALT), meat extract (MEAT), peptone (PPT), sodium acetate (SA), trypticase soy broth (TRP), and yeast extract (YST). Plotted values are the mean of replicate measurements and error bars represent one SD.

Planktonic growth and biofilm formation of each test strain were evaluated using various carbon and energy sources in order to identify the optimal nutrient conditions for growth and biofilm formation over 1, 3, and 7 days, with 7 days representing longer-term biofilm establishment. *Bacillus subtilis* 168 displayed significantly different planktonic growth after 7 days across the different culture conditions (Fig. 1, Table S3), with the greatest planktonic growth obtained using 0.25% w/v meat extract. Significantly different biofilm responses were also observed. 0.50% w/v meat extract, which resulted in the greatest biofilm formation of all nutrients at day 7, was identified as the “optimal” culture condition for biofilm formation in this strain.

**Figure 2.**
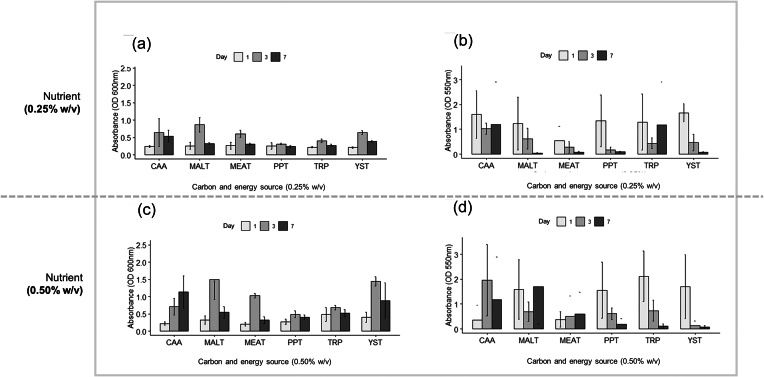
*Fusarium solani* (Martius) Saccardo planktonic growth and biofilm formation measured by absorbance (600 and 550 nm, respectively) at various incubation periods (day 1, 3, and 7) to compare different carbon/energy sources and concentrations. Day 7 biofilm formation results were then examined to identify the “optimal” nutrient which displayed significant and long-term biofilm formation. (a) *F. solani* planktonic growth using 0.25% w/v nutrient concentrations. (b) *F. solani* biofilm response using 0.25% w/v nutrient concentrations. (c) *F. solani* planktonic growth using 0.50% w/v nutrient concentrations. (d) *F. solani* biofilm response using 0.50% w/v nutrient concentrations. Abbreviations: casamino acids (CAA), ethylene glycol (EG), malt extract (MALT), meat extract (MEAT), peptone (PPT), sodium acetate (SA), trypticase soy broth (TRP), and yeast extract (YST). Plotted values are the mean of replicate measurements and error bars represent one SD.

*Fusarium solani* (Fig. 2, Table S3) displayed significantly different day 7 planktonic growth across the various carbon and energy sources. Additionally, significantly different biofilm responses were observed at day 7 when cultured in 0.25% w/v nutrients. However, the 0.50% w/v nutrient concentrations displayed highly variable biofilm responses with no significant difference between carbon and energy sources. For this species, malt extract (0.50% w/v) was identified as the “optimal” nutrient which invoked the greatest day 7 biofilm

**Figure 3.**
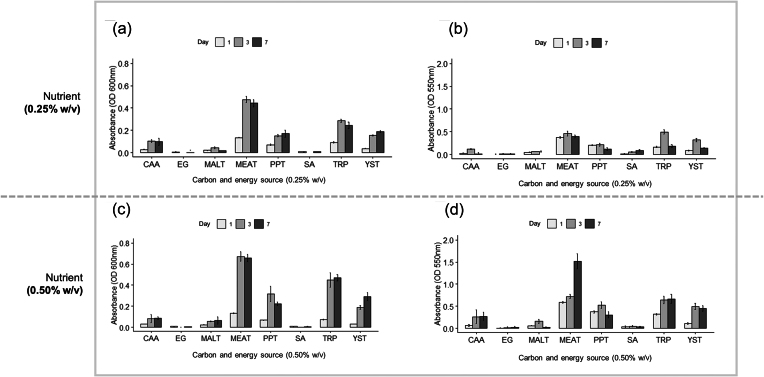
*Ideonella sakaiensis* 201-F6 planktonic growth and biofilm formation measured by absorbance (600 and 550 nm, respectively) at various incubation periods (day 1, 3, and 7) to compare different carbon/energy sources and concentrations. Day 7 biofilm formation results were then examined to identify the “optimal” nutrient which displayed significant and long-term biofilm formation. (a) *I. sakaiensis* 201-F6 planktonic growth using 0.25% w/v nutrient concentrations. (b) *I. sakaiensis* 201-F6 biofilm response using 0.25% w/v nutrient concentrations. (c) *I. sakaiensis* 201-F6 planktonic growth using 0.50% w/v nutrient concentrations. (d) *I. sakaiensis* 201-F6 biofilm response using 0.50% w/v nutrient concentrations. Abbreviations: casamino acids (CAA), ethylene glycol (EG), malt extract (MALT), meat extract (MEAT), peptone (PPT), sodium acetate (SA), trypticase soy broth (TRP), and yeast extract (YST). Plotted values are the mean of replicate measurements and error bars represent one SD.

*Ideonella sakaiensis* 201-F6 (Fig. 3, Table S3) exhibited significantly different planktonic and biofilm growth in the various culture conditions. Meat extract invoked the greatest planktonic response at both 0.25% w/v and 0.50% w/v when compared against the other nutrient sources. Notably, meat extract displayed a rapid planktonic response which plateaued between days 3 and 7. Meat extract (0.50% w/v) also exhibited the most significant biofilm response at day 7 and was therefore identified as the “optimal” nutrient. *I. sakaiensis* did not display any significant planktonic growth or biofilm formation in ethylene glycol or sodium acetate at 0.25% or 0.5% w/v concentrations.

**Figure 4.**
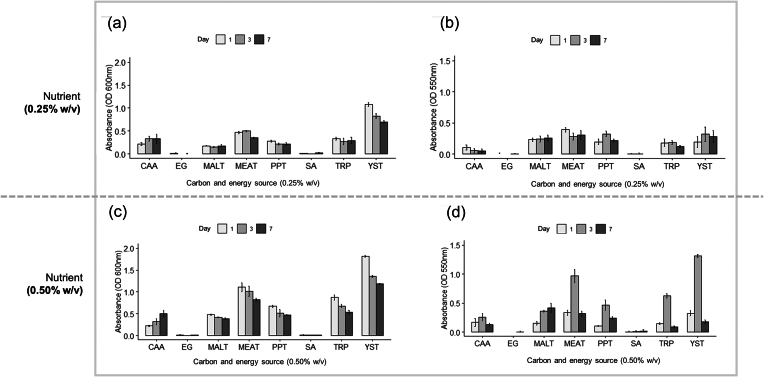
*Pseudomonas putida* KT2440 planktonic growth and biofilm formation measured by absorbance (600 and 550 nm, respectively) at various incubation periods (day 1, 3 and 7) to compare different carbon/energy sources and concentrations. Day 7 biofilm formation results were then examined to identify the “optimal” nutrient which displayed significant and long-term biofilm formation. (a) *P. putida* KT2440 planktonic growth using 0.25% w/v nutrient concentrations. (b) *P. putida* KT2440 biofilm response using 0.25% w/v nutrient concentrations. (c) *P. putida* KT2440 planktonic growth using 0.50% w/v nutrient concentrations. (d) *P. putida* KT2440 biofilm response using 0.50% w/v nutrient concentrations. Abbreviations: casamino acids (CAA), ethylene glycol (EG), malt extract (MALT), meat extract (MEAT), peptone (PPT), sodium acetate (SA), trypticase soy broth (TRP), and yeast extract (YST). Plotted values are the mean of replicate measurements and error bars represent one SD.

*Pseudomonas putida* KT2440 (Fig. 4, Table S3) displayed significantly different planktonic and biofilm growth when cultured in the various nutrient conditions (day 7). Yeast extract resulted in the highest planktonic responses for both 0.25% w/v and 0.50% w/v concentrations. However, malt extract (0.5% w/v) was identified as the “optimal” biofilm nutrient, which maintained a significant longer-term biofilm at day 7. *P. putida* KT2440 also displayed no significant planktonic growth or biofilm formation in ethylene glycol or sodium acetate at 0.25% w/v or 0.50% w/v concentrations.

**Figure 5.**
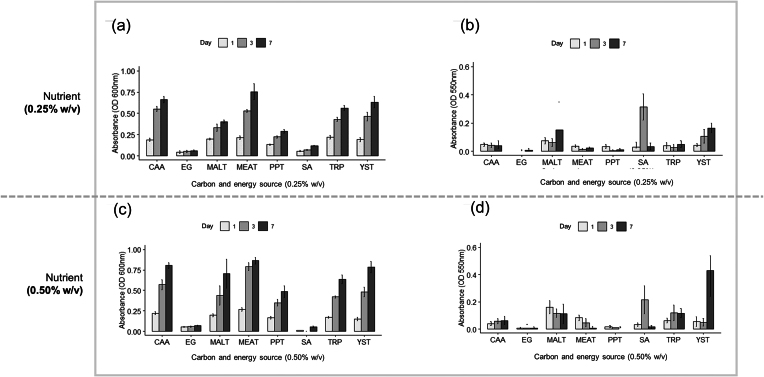
*Rhodococcus ruber* C208 planktonic growth and biofilm formation measured by absorbance (600 and 550 nm, respectively) at various incubation periods (day 1, 3 and 7) to compare different carbon/energy sources and concentrations. Day 7 biofilm formation results were then examined to identify the “optimal” nutrient, which displayed significant and long-term biofilm formation. (a) *R. ruber* C208 planktonic growth using 0.25% w/v nutrient concentrations. (b) *R. ruber* C208 biofilm response using 0.25% w/v nutrient concentrations. (c) *R. ruber* C208 planktonic growth using 0.50% w/v nutrient concentrations. (d) *R. ruber* C208 biofilm response using 0.50% w/v nutrient concentrations. Abbreviations: casamino acids (CAA), ethylene glycol (EG), malt extract (MALT), meat extract (MEAT), peptone (PPT), sodium acetate (SA), trypticase soy broth (TRP), and yeast extract (YST). Plotted values are the mean of replicate measurements and error bars represent one SD.

*Rhodococcus ruber* C208 (Fig. 5, Table S3) displayed significant planktonic growth across multiple carbon and energy sources, including casamino acids, malt extract, meat extract, peptone, tryptone, and yeast extract. Specifically, meat extract (0.50% w/v) exhibited the most significant day 7 planktonic response. Yeast extract (0.5% w/v) was identified as the “optimal” biofilm nutrient, as it resulted in the greatest long-term biofilm formation at 7 days. Notably, *R. ruber* displayed some planktonic growth with ethylene glycol, a PET monomer. Whilst it did not display significant planktonic growth in sodium acetate, substantial biofilm formation was observed by day 3, at both 0.25% w/v and 0.50% w/v concentrations.

### Isolation of plastic colonizers from plastic-contaminated environmental samples

After 42 days, plastic films (HDPE, LDPE, and PET) incubated with three environmental samples (anaerobic digestate, contaminated compost, and landfill leachate) yielded eight bacterial isolates which had successfully colonized their surfaces via biofilm formation. The isolates were then subjected to identification using a MALDI-TOF microbial identification system (Table 3). Plastic films incubated in anaerobic digestate resulted in the isolation of a single unique bacterial colonizer from each plastic type: *Proteus vulgaris* (from PET), *Lysinibacillus* sp. (from LDPE), and *Bacillus proteolyticus* (from HDPE). Films incubated in landfill leachate resulted in the isolation of a single species, (*Proteus vulgaris*), which was found on both PET and HDPE. From contaminated compost, three bacteria sharing a common genus (*Bacillus*) were isolated across all three plastics, with *B. cereus* identified as a primary colonizer of both LDPE and HDPE films. Within environmental samples, different bacterial strains were found to colonize each unique plastic polymer. For example, within the anaerobic digestate sample, only *Lysinibacillus* sp. was recovered from LDPE films, whereas only *Bacillus* sp. was recovered on HDPE from the same sample. Additionally, comparison between environmental samples identified common bacterial genera across these discrete microbial ecosystems. For example, *Bacillus* spp. were identified as the primary colonizers of HDPE films in both anaerobic digestate and contaminated compost after 42 days. Furthermore, *P. vulgaris* displayed an ability to colonize PET films within anaerobic digestate and landfill leachate, both vastly different environmental samples.

**Table 3.** Summary of environmental isolate identification using MALDI-TOF mass spectrometry.

Isolate (No.)	Plastic film	Environmental sample	Identification (genus/species)	Score	Confidence level
**E1**	PET	Anaerobic digestate	*Proteus vulgaris*	9.478	Species
**E2**	LDPE	Anaerobic digestate	*Lysinibacillus* sp.	8.619	Genus
**E3**	HDPE	Anaerobic digestate	*Bacillus* sp.	5.670	Unreliable*
**E4**	PET	Landfill leachate	*Proteus vulgaris*	9.508	Species
**E5**	HDPE	Landfill leachate	*Proteus vulgaris*	9.553	Species
**E6**	PET	Contaminated compost	*Bacillus* sp.	6.648	Genus
**E7**	LDPE	Contaminated compost	*Bacillus* sp.	8.566	Genus
**E8**	HDPE	Contaminated compost	*Bacillus cereus*	9.035	Species

* The confidence score for isolate E3 was below the cut-off value for “reliable” identification (6.0), necessitating further analysis through 16S rRNA gene sequencing (Supplementary methods). Following a BLAST search of the resulting sequence, the closest match was identified as *B. proteolyticus* strain W2 (Genbank accession no. MH921605.1), with 99.67% sequence similarity.

## Discussion

Overcoming the initial surface colonization barrier through biofilm formation is a fundamental prerequisite to plastic biodegradation (Plakunov et al. 2020). During this study, microtiter assays were used to evaluate the planktonic growth and biofilm responses of five known PET and LDPE plastic-degrading strains to diverse nutrient conditions. *Bacillus subtilis* 168*, F. solani* (Martius) Saccardo*, I. sakaiensis* 201-F6*, P. putida* KT2440, *and R. ruber* C208 all displayed significant differences (*P* < 0.05) in both long-term (Day 7) planktonic growth and biofilm formation when cultured in various carbon and energy sources. Furthermore, “optimal” biofilm enhancing nutrient conditions were identified for each strain: *B. subtilis* 168 (0.5% w/v meat extract), *F. solani* (0.5% w/v malt extract), *I. sakaiensis* 201-F6 (0.5% w/v meat extract), *P. putida* KT2440 (0.5% w/v malt extract), and *R. ruber* C208 (0.5% w/v yeast extract). Notably, meat, malt, and yeast extracts caused substantial biofilm responses across all strains, potentially attributed to the variety of carbon, nitrogen and growth factors they contain, which directly influence microbial metabolism (Wijesinghe et al. 2019). For example, yeast extract contains a diverse portfolio of essential amino acids, vitamins, and trace elements, with over 4000 individual oligopeptides (Proust et al. 2019). Alongside carbon utilization, nitrogen content has been indicated to significantly influence biofilm composition (Ramos et al. 2023).

Independent strains displayed the variable relationship between planktonic growth and biofilm formation. For example, *B. subtilis* 168 displayed the greatest planktonic growth in 0.5% (w/v) malt extract, whilst long-term biofilm formation was invoked by 0.5% (w/v) meat extract. Consistently, Koim-Puchowska et al. (2021) reported the impact of carbon source and culture conditions on *B. subtilis natto* BS19 biomass and surfactin biosynthesis, a key quorum sensing (QS) signalling molecule associated with biofilm formation. However, the highest surfactin yield was produced using both starch (carbon source) and yeast extract (nitrogen source) (Koim-Puchowska et al. 2021).

*Rhodococcus ruber* C208 exhibited substantial planktonic responses in a variety of carbon and energy sources, suggesting a more generalist and versatile metabolism. Zampolli et al. (2022) outlined that the *Rhodococcus* genus displays remarkable metabolic capabilities, especially under environmental stress (Zampolli et al. 2022). However, the most significant biofilm formation (day 7) was a result of growth in yeast extract (0.5% w/v). Contrastingly, *P. putida* KT2440 displayed the greatest long-term biofilm formation when cultured using malt extract (0.5% w/v). *F. solani* also illustrated the greatest biofilm formation in 0.5% (w/v) malt extract, perhaps suggesting similar biofilm responses between bacteria and fungi. However, *F. solani* also displayed the greatest variance in biofilm responses between the nutrient conditions, which is likely due to the disparate growth patterns of filamentous fungi. Shay et al. (2022) outlined the highly transient nature of mature *Fusarium graminearum* biofilms, which displayed weaker polystyrene attachment after 48 hours. Therefore, alternatives to 96-well microtiter assays should be explored to investigate filamentous fungal biofilms.

In contrast, *I. sakaiensis* 201-F6 displayed analogous planktonic and biofilm responses in meat extract (0.5% w/v), suggesting a greater direct relationship between planktonic growth and biofilm establishment in this species. Furthermore, *I. sakaiensis* 201-F6 planktonic growth plateaued between day 3 and 7, while biofilm development continued to increase until day 7. These findings coincide with the lifestyle transition of planktonic cells into biofilm communities as a long-term survival strategy in response to reduced nutrient availability (Mishra et al. 2022). Importantly, the identification of meat extract as a biofilm enhancing nutrient could potentially be applied in biodegradation studies. For example, the original PET biodegradation study using *I. sakaiensis* 201-F6 utilized polypeptone (1.0% w/v) and yeast extract (0.2% w/v) as supplemental carbon sources in the culture medium to facilitate biodegradation (Yoshida et al. 2016).

Regarding intermediate metabolism, intracellular ethylene glycol metabolic pathways have been identified in both *I. sakaiensis* 201-F6 and *P. putida* KT2440 (Yoshida et al. 2016, Brandenberg et al. 2022). However, our investigation displayed no significant planktonic growth in ethylene glycol at the two concentrations tested. Notably, whilst this study investigated the wild-type *P. putida* KT2440 strain, previous research has exploited metabolic engineering and adaptive library evolution to increase ethylene glycol metabolism in this species (Franden et al. 2018, Li et al. 2019, Brandenberg et al. 2022). For example, Li et al. (2019) reported increased *P. putida* KT2440 growth on ethylene glycol via mutations to the transcriptional regulator PP_2046 and porin encoded by PP_2662. Interestingly, this study demonstrated planktonic growth of *R. ruber* C208 in ethylene glycol at both concentrations tested. Therefore, these findings suggest that *R. ruber* C208 possesses metabolic pathways for the metabolism of ethylene glycol, which could be further explored in the context of alleviating inhibition by intermediates. Similarly, previous transcriptome analysis of *R. ruber* C208 by Gravouil et al. (2017) revealed upregulation of alkane degradation enzymes and fatty acid β-oxidation pathways in the presence of PE. Excitingly, current plastic biodegradation studies using *R. ruber* C208 primarily focus on LDPE with limited discussion of PET.

Reported biodegradation rates of plastic are extremely slow, even in optimized conditions (Jacquin et al. 2019). Overall, the wider literature confirms the importance and relationship between increased biofilm formation and accelerated plastic biodegradation (Tribedi and Sil 2013, Kotova et al. 2021, Howard and McCarthy 2023), highlighting the need for studies of the interactions between microorganisms and the plastics they degrade. In this study, we identified specific biofilm-enhancing nutrient conditions for five known plastic-degrading strains to potentially enhance biodegradation rates in subsequent studies. However, as biofilm development is multifactorial, it is important to also explore additional factors such as alternative medium components, initial inoculum density, EPS composition, QS signalling, and the effects of accumulated waste products (Fathollahi and Coupe 2021, Gao et al. 2021, Nishikawa and Kobayashi 2021). Furthermore, the application of multi-omic approaches could enable insights into the interactions between biofilm-associated genes, transcripts, enzymes, and metabolic pathways (Amobonye et al. 2021, Chattopadhyay 2022).

Investigating microbial populations within plastic polluted ecosystems is essential if novel degradative capabilities are to be identified (Wallbank et al. 2022). Microplastic polluted environments were screened using a culture-based approach with untreated plastic films (HDPE, LDPE, and PET), which resulted in the identification of eight bacterial isolates (Table 3) which had successfully colonized their surfaces after 42 days. These bacterial isolates were from three main genera: *Bacillus, Lysinibacillus*, and *Proteus*. The colonization of the plastic films by these isolates demonstrates that they were able to overcome the important initial barrier of surface hydrophobicity of these synthetic polymers. According to Maclean et al. (2021), plastics create selective habitats that support distinct microbial communities, which is in agreement with our own findings. This study indicated that the three discrete plastic types (HDPE, LDPE, and PET) provided different ecological niches for unique bacterial colonizers to exploit. For example, *Lysinibacillus* was the sole isolate recovered from LDPE films incubated with the anaerobic digestate sample, whereas *B. proteolyticus* was the sole isolate obtained on HDPE from the same sample. Contrastingly, within the contaminated compost sample, *Bacillus cereus* was isolated from both LDPE and HDPE samples. Furthermore, the association between *Lysinibacillus* and *Bacillus* spp. in PE biofilm formation is supported by wider literature and the Plastic Microbial Biodegradation Database (PMBD) (Gambarini et al. 2022). Jeon et al. (2021) described the isolation of *Lysinibacillus* sp. JJYO216 from a soil grove, which degraded LDPE by 9% over 26 days. Similarly, Sangeetha Devi et al. (2019) investigated the marine microbiome by screening 248 isolates for HDPE degradation efficiency, identifying *Bacillus* spp. as the most potent HDPE biodegraders.

Interestingly, this study indicated that the facultative anaerobe *P. vulgaris* was the primary colonizer of PET films in two different environmental samples (anaerobic digestate and landfill leachate), potentially suggesting shared microbial populations between agricultural and municipal waste streams. Importantly, these findings correspond with the engineered nature of the landfill ecosystem; landfill leachate percolates through the layers of the landfill and is collected using an impermeable HDPE base liner (Meyer-Dombard et al. 2020). Specifically, *Proteus* spp. have been associated with lipase production and petroleum hydrocarbon utilization in contaminated soil, oil sludge, and wastewater (Kim et al. 1996, Ke et al. 2021). However, existing literature focuses on *P. vulgaris* in the context of PE degradation. This study indicated *P. vulgaris* as a novel PET colonizer, highlighting the potential that it may possess uncharacterized PET metabolic pathways. The functional potential of these and other bacterial strains should be explored to uncover novel enzymatic and metabolic pathways linked to plastic colonization and biodegradation.

## Conclusion

Microbial biodegradation of single-use plastic represents an emerging approach to plastic pollution. A major bottleneck of microbial plastic biodegradation is caused by the low bioavailability and highly recalcitrant properties of synthetic polymers, which prevent initial surface attachment. This study aimed to enhance the biofilm establishment of existing PE and PET degrading strains: *B. subtilis* 168, *F. solani* (Martius) Saccardo, *I. sakaiensis* 201-F6, *P. putida* KT2440 and *R. ruber* C208. Microtiter assay results displayed the variable relationship between planktonic growth and biofilm formation of each strain when cultured in various carbon and energy sources. Importantly, the results enabled the successful identification of long-term biofilm enhancing nutrient conditions with translational potential for improved plastic biodegradation. Interestingly, *R. ruber* C208 exhibited planktonic growth in ethylene glycol (a PET monomer) as a sole carbon source, suggesting the presence of intrinsic ethylene glycol metabolic pathways. Separately, environmental screening of microplastic polluted ecosystems enabled the identification of eight bacterial strains adept at LDPE, HDPE, and PET surface colonization. Furthermore, this study demonstrated that discrete plastic types create different ecological niches for unique bacterial colonizers to exploit. Excitingly, this study revealed *P. vulgaris* as a PET colonizer, a species that was previously undescribed in the context of PET biodegradation. Our findings underscore the importance of considering plastic colonization by microbial biofilms in the context of their biodegradation.

## Supplementary Material

qvae012_Supplemental_File

## Data Availability

The data underlying this article will be shared on reasonable request to the corresponding author.
