# Supplementary material for: Strategies for biofilm optimization of plastic-degrading microorganisms and isolating biofilm formers from plastic-contaminated environments
Source: Sustain Microbiol. 2024 May 17;1(1):qvae012. doi: 10.1093/sumbio/qvae012 (PMC13375355; doi:10.1093/sumbio/qvae012)
Supplement: Supplementary file 1 [file qvae012_Supplemental_File.docx]

**Supplementary methods: identification of isolate E3 by 16S rRNA gene sequencing**

Pure colonies were added to 500 µL nuclease-free water, vortexed for 15 seconds, and heated to 99 ˚C for 10 min to lyse cells. The cell lysate (1 µL) was added to 49 µL of reaction mixture which consisted of 25 µL DreamTaq Green PCR Master Mix (Thermo Scientific), 5 µL each of 2.5 µM 27F universal forward primer (5’-AGAGTTTGATCMTGGCTCAG-3’) and 2.5 µM 1492R universal reverse primer (5’-GGTTACCTTGTTACGACTT-3’), and 14 µL nuclease-free water. DNA was amplified under the following conditions: 30 cycles of 94 ˚C for 45 s, 55 ˚C for 45 s, and 72 ˚C for 60 s, followed by a final extension at 72 ˚C for 10 min, and storage at 4 ˚C. PCR products were analysed by loading 5 µL of each reaction mixture into an agarose gel (1% w/v agarose in TAE buffer; 10V/cm for 1 h) containing GelRed Nucleic Acid Gel Stain (Biotium, USA). FastRuler Low Range DNA Ladder (Thermo Scientific) and a negative control sample were also loaded into the gel. Successful amplicons (approx. 1500 bp) were identified and purified using a GeneJET PCR purification kit (Thermo Scientific) on the remaining 45 µL of the reaction mixtures.

Purified PCR products were quantified using a Qubit HS DNA assay kit (Thermo Scientific) and adjusted to 10 ng/µL with nuclease-free water. These were then separately mixed with 27F and 1492R primers and sequenced by Eurofins Genomics (Cologne, DE) using the Sanger sequencing platform. The resulting forward and reverse reads were assembled into a consensus sequence using the CLUSTALW multiple sequence alignment program. The sequence was then analysed by comparison to the sequences available within the National Center for Biotechnology Information (NCBI) GenBank database, using the BLASTn tool.

**Table S1.** Media and culture conditions for each strain and environmental isolates.

| **Strain** | **Media Components** | **Temperature (˚C)** |
| --- | --- | --- |
| *Bacillus subtilis 168* | DSMZ Medium 1, containing: peptone (5.0 g L^-1^) and meat extract (3.0 g L^-1^) | 30 |
| *Fusarium solani* (Martius) Saccardo | DSMZ Medium 90, containing: malt extract (30.0 g L^-1^) and soya peptone (3.0 g L^-1^) |  |
| *Ideonella sakaiensis* 201-F6 | Modified NBRC medium containing: peptone (10.0 g L^-1^), yeast extract (2.0 g L^-1^) and MgSO_4_.7H_2_O (2.0 g L^-1^) |  |
| *Pseudomonas putida* KT2440 | DSMZ Medium 1, containing: peptone (5.0 g L^-1^) and meat extract (3.0 g L^-1^) |  |
| *Rhodococcus ruber* C208 | DSMZ Medium 535, containing: Trypticase soy broth (30.0 g L^-1^) |  |
| Environmental Isolates | Luria-Bertani medium was used to maintain environmental isolates, containing: peptone (10.0 g L^-1^), yeast extract (5.0 g L^-1^), NaCl (5.0 g L^-1^). | 28 |

**Table S2.** Equipment and reagents list.

| **Equipment / Reagent** | **Supplier / Manufacturer / Item Code** |
| --- | --- |
| Microtiter assay plate | Thermo Fisher NUNC Microwell 96-Well Plate |
| Microplate reader | BMG Labtech CLARIOstar plate reader |
| Analytical balance | Sartorius fine balance |
| MALDI-TOF mass spectrometer | Autobio Autof ms1600 |
| Crystal violet | Sigma-Aldrich® (C0775-25G) |
| Acetic Acid | VWR Chemicals (CAS No. 64-19-7) |
| Sodium dodecyl sulphate | Sigma-Aldrich® (71736-100ML) |
| Sodium acetate | Honeywell \| Fluka (CAS No.127-09-3) |
| Ethylene glycol | Sigma-Aldrich® (102466-2.5L) |
| Meat extract | Sigma-Aldrich® Fluka (70164-500G) |
| Malt extract | Sigma-Aldrich® (70167-500G) |
| Yeast extract | Thermo Fisher - Oxoid (Code: LP0021) |
| Bacteriological peptone | Thermo Fisher - Oxoid (Code: LP0034) |
| Trypticase Soy Broth | VWR Chemicals (Catalogue No. ICNA0215715283) |
| Bacto™ Casamino acids | Thermo Fisher (Catalogue No. 223050) |
| Sodium chloride | Sigma-Aldrich® (S3014-500G) |
| Magnesium sulfate heptahydrate | Honeywell \| Fluka (Cat. 13142-1KG) |

**CONTROLS**

**Figure S1.** Microtiter assay plate layout displaying test wells (yellow) and control wells (blue) and excluded rows A and H (red).

**Table S3.** Day 7 planktonic growth and biofilm response statistical analysis.

| **Microorganism** | **Environmental Condition** | **Day 7 Statistical Comparisons** |
| --- | --- | --- |
| ***B. subtilis* 168** | Planktonic growth (0.25% w/v nutrients) | Kruskal-Wallis chi-squared = 45.0653, df = 7, p-value = 1.328e-07 |
|  | Planktonic growth (0.50% w/v nutrients) | Kruskal-Wallis chi-squared = 44.9882 , df = 7, p-value = 1.375e-07 |
|  | Biofilm response (0.25% w/v nutrients) | Kruskal-Wallis chi-squared = 23.6317 , df = 7, p-value = 0.0008817 |
|  | Biofilm response (0.50% w/v nutrients) | Kruskal-Wallis chi-squared = 37.5608 , df = 7, p-value = 3.672-06 |
| ***F. solani* (Martius) Saccardo** | Planktonic growth (0.25% w/v nutrients) | Kruskal-Wallis chi-squared = 28.2956 , df = 5, p-value = 8.973e-07 |
|  | Planktonic growth (0.50% w/v nutrients) | Kruskal-Wallis chi-squared = 16.7702 , df = 5, p-value = 1.296e-05 |
|  | Biofilm response (0.25% w/v nutrients) | Kruskal-Wallis chi-squared = 11.382 , df = 5, p-value = 0.04 |
|  | Biofilm response (0.50% w/v nutrients) | Kruskal-Wallis chi-squared = 2.0516 , df = 5, p-value = 0.842 |
| ***I. sakaiensis* 201-F6** | Planktonic growth (0.25% w/v nutrients) | Kruskal-Wallis chi-squared = 44.7157, df = 7, p-value = 1.553e-07 |
|  | Planktonic growth (0.50% w/v nutrients) | Kruskal-Wallis chi-squared = 45.4309, df = 7, p-value = 1.128e-07 |
|  | Biofilm response (0.25% w/v nutrients) | Kruskal-Wallis chi-squared = 44.394, df = 7, p-value = 1.792e-07 |
|  | Biofilm response (0.50% w/v nutrients) | Kruskal-Wallis chi-squared = 43.863 , df = 7, p-value = 2.272e-07 |
| ***P. putida* KT2440** | Planktonic growth (0.25% w/v nutrients) | Kruskal-Wallis chi-squared = 42.3193, df = 7, p-value = 4.513e-07 |
|  | Planktonic growth (0.50% w/v nutrients) | Kruskal-Wallis chi-squared = 44.7777 , df = 7, p-value = 1.51e-07 |
|  | Biofilm response (0.25% w/v nutrients) | Kruskal-Wallis chi-squared = 41.8489 , df = 7, p-value = 5.56e-07 |
|  | Biofilm response (0.50% w/v nutrients) | Kruskal-Wallis chi-squared = 45.0608 , df = 7, p-value = 1.331e-07 |
| ***R. ruber* C208** | Planktonic growth (0.25% w/v nutrients) | Kruskal-Wallis chi-squared = 44.537, df = 7, p-value = 1.682e-07 |
|  | Planktonic growth (0.50% w/v nutrients) | Kruskal-Wallis chi-squared = 40.079 , df = 7, p-value = 1.216e-06 |
|  | Biofilm response (0.25% w/v nutrients) | Kruskal-Wallis chi-squared = 30.384, df = 7, p-value = 8.073e-05 |
|  | Biofilm response (0.50% w/v nutrients) | Kruskal-Wallis chi-squared = 41.364 , df = 7, p-value = 6.893e-07 |
